# Perceptions of parents and healthcare professionals regarding minimal invasive tissue sampling to identify the cause of death in stillbirths and neonates: a qualitative study protocol

**DOI:** 10.1186/s12978-018-0626-0

**Published:** 2018-10-22

**Authors:** Anam Feroz, Mohsina Noor Ibrahim, Shiyam Sunder Tikmani, Sayyeda Reza, Zahid Abbasi, Jamal Raza, Haleema Yasmin, Khadija Bano, Afia Zafar, Elizabeth M. McClure, Robert L. Goldenberg, Sarah Saleem

**Affiliations:** 1 Department of Community Health Sciences, The Aga Khan University, Stadium Road, PO Box 3500, Karachi, 74800 Pakistan; 2 National Institute of Child Health, Karachi, Pakistan; 3 Department of Obstetrics and Gynecology, Jinnah Post-graduate Medical Center, Karachi, Pakistan; 4 Department of Pathology & Laboratory Medicine, The Aga Khan University, Stadium Road, PO Box 3500, Karachi, 74800 Pakistan; 5 RTI International, Durham, USA; 6 Department of Obstetrics and Gynecology, Columbia University, New York, USA

**Keywords:** Minimal invasive tissue sampling, Cause of death, Neonates, Still-birth, Qualitative study, Parents, Healthcare professionals, Full-autopsy

## Abstract

**Background:**

Globally, around 2.6 million neonatal deaths occur world-wide every year and the numbers of stillbirths is almost similar. Pakistan is ranked among the highest countries in the world for neonatal mortality. In 2016, for every 1000 babies born in Pakistan, 46 died before the end of the first month of life. Also, Pakistan had the highest rate of stillbirths (43.1/1000 births) in 2015. To meet sustainable development (SDG) targets of reducing neonatal mortality and stillbirths, it is essential to gain understanding about the causes of neonatal death and stillbirths. In Pakistan, full autopsies are conducted only in medico-legal cases and are very rarely performed to identify a definitive cause of death (CoD) and because of cost and insufficient staff are generally not feasible. Recently, minimally invasive tissue sampling (MITS) has been used to determine CoD in neonates and stillbirths as it addresses some of the socio-cultural and religious barriers to autopsy. However, it is not known how families and communities will perceive this procedure; therefore, exploring family and healthcare professionals’ perceptions regarding MITS is essential in determining acceptable and feasible approaches for Pakistan.

**Methods:**

The study will employ an exploratory qualitative research design. The study will be conducted at the National Institute of Child Health (NICH) hospital of Karachi. The data collection method will consist of key-informant interviews (KIIs) and focus group discussions (FGDs). FGDs will be conducted with the families and relatives of newborns who are visiting the outpatient department (OPD) and well-baby clinics of NICH hospital. KIIs will be conducted with the NICH - medical director, healthcare providers, professionals involved in proceedings related to death and dying, religious leaders, health sector representatives from the government, public health experts, maternal and child health (MCH) specialists, obstetricians and neonatologists and experts from the bioethics committee. Study data will be analyzed using NVivo 10 software.

**Discussion:**

The research will help explore specific cultural, religious and socio-behavioral factors that may increase or decrease the acceptability of MITS for identifying COD in neonates and stillbirths. The findings of the qualitative study will provide a better understanding of parents’ and healthcare professionals’ attitudes towards the use of MITS on neonatal deaths and stillborns.

## Plain English summary

Pakistan is ranked among the highest countries in the world for neonatal mortality. In 2016, for every 1000 babies born in Pakistan, 46 died before the end of the first month of life. Also, Pakistan had the highest rate of stillbirths (43.1/1000 births) in 2015. To meet sustainable development (SDG) targets of reducing neonatal mortality and stillbirths, it is essential to gain understanding about the causes of neonatal death and stillbirths. Recently, MITS has been used to determine CoD in neonates and stillbirths as it addresses some of the socio-cultural and religious barriers to autopsy. However, it is not known how families and communities will perceive this procedure. Therefore, the purpose of this formative research is to explore the facilitating and inhibitory factors perceived by communities and healthcare professionals for the implementation of the MITS procedure.

The study will employ an exploratory qualitative research design. The study will be conducted at the National Institute of Child Health (NICH) hospital of Karachi. The data collection method will consist of key-informant interviews (KIIs) and focus group discussions (FGDs). Study data will be analyzed using NVivo 10 software.

This qualitative study will provide a thorough insight into the views of families and healthcare professionals, towards the use of MITS. The study will also highlight how a less invasive autopsy can address some of the barriers, such as delay in carrying out funeral practices and concerns regarding organ and tissue removal, which are particularly significant for some cultural and religious groups.

## Background

There are approximately 2.6 million neonatal deaths world-wide every year and the number of stillbirths is almost similar. The number of neonatal deaths worldwide has declined from 5.1 million in 1990 to 2.6 million in 2016. This decline has been slower when compared to the under-5 mortality rates, i.e. 49% compared with 62%. In the first month of life, nearly half of all neonatal deaths take place in the first 24 h, and up to 75% of all neonatal deaths occur in the first week of life [1]. As per the recent UNICEF report, Pakistan is ranked among the highest countries in the world with regard to neonatal mortality. In 2016, for every 1000 babies born in Pakistan, 46 died before the end of the first month of their life. Also, Pakistan had the highest rate for stillbirths (43.1/1000 births) in 2015 making it the worst performer amongst 186 countries [2]. Therefore, Pakistan is considered to be one of the riskiest places in the world for childbirth. In order to reach the sustainable development (SDG) targets for neonatal mortality and stillbirth rates of 12 deaths per 1000 live births by 2030 [3], it is very important to identify the causes of death.

In low-middle-income countries (LMICs), infants often die without being cared for by a skilled healthcare professional, without any documentation from a medical examiner and are usually buried without conducting any cause of death (CoD) investigation [4]. Determining the causes of neonatal death and stillbirth in healthcare facilities and in the communities is crucial for several reasons. CoD determination is important to reveal the actual cause of neonatal death and stillbirth. CoD investigations are important to resolve uncertainties in global disease estimations. Lastly, the right information about the cause of neonatal deaths and stillbirths will help develop effective public health programs and will allow public health policymakers to make informed decisions for allocating health care resources [5–7].

In LMICs, full autopsies on neonates and stillbirths are rarely conducted to identify a definitive CoD and are unlikely to be feasible due to both resource constraints and acceptability issues. In countries like Pakistan, full-autopsies are not performed due to cultural, financial, religious, and physical barriers, except for medico-legal cases [7, 8]. The minimally invasive tissue sampling (MITS) procedure is now being used to address the socio-cultural and religious barriers [9]. The MITS procedure involves extracting tissue specimens from a predefined set of organs and using that tissue for histopathologic examinations and organism identification. The methodology offers the possibility of gathering critical missing data to determine the causes of death in neonates and stillbirths. MITS is potentially quicker, less expensive, more acceptable and markedly less invasive, and has great potential to determine the CoD nearly as accurately as a full autopsy [10]. However, it is not known how families, communities and healthcare professionals will perceive this procedure or how they will decide whether or not to consent to a post-mortem needle biopsy as it still involves technical and cultural challenges [5].

Implementation of MITS procedures in areas where post-mortem procedures have been seldom utilized, such as in a Muslim country like Pakistan, requires an understanding of what is culturally and religiously is acceptable and feasible [6, 11]. Furthermore, understanding how, when and by whom, and in which context grieving relatives of a deceased neonate or stillbirth should be approached to seek permission to perform such procedures is critical. Very few studies have explored facilitators and barriers to the MITS procedure [12]. Exploring family and healthcare professionals’ perceptions and views regarding the MITS procedure is essential in defining best practice and determining acceptable and feasible approaches [6].

### Rationale

As the MITS procedure is performed on the body of a recently deceased neonate or stillbirth, a number of complex factors including religious beliefs, cultural norms, financial limitations and ethical constraints inevitably arise and may make MITS difficult to execute in LMICs, and Pakistan is not different. Widespread uptake and acceptability of MITS will require a thorough understanding of the ethical issues, cultural and religious norms and practices to determine the feasibility of MITS prior to implementation [4]. For example, beliefs about death and the afterlife, opposition to and concerns about body disfigurement, difficulties in obtaining consent from grieving families, inadequate involvement/endorsement of community leaders, lack of community awareness, suspicion of researchers, and burial practices are some of the factors underlying MITS refusal. Understanding these kinds of family perceptions and healthcare professional concerns regarding MITS will be essential to increase community participation and acceptability.

### Study purpose

The purpose of this formative research is to explore the facilitating and inhibitory factors perceived by communities and healthcare professionals for the implementation of the MITS procedure.

### Study objectives

- To explore and understand families’ perceptions and views regarding premature births, stillbirths and neonatal deaths and its related causes.
- To explore cultural, social and religious norms and conduct around deaths (neonatal deaths/stillbirths)
- To explore families’ and healthcare professionals’ willingness to know the cause of death for neonates and stillbirths
- To examine families’ and healthcare providers’ attitudes towards the MITS procedure
- To identify perceived facilitators and barriers for the implementation of MITS procedure among families and healthcare professionals

## Methods

### Study design

This formative research will employ an exploratory qualitative research design using semi-structured interviews and a purposive sampling approach. The data collection methods for this formative research will involve key-informant interviews (KIIs) and focus group discussions (FGDs). The aim of the FGDs and KIIs is to explore and understand the acceptability of MITS procedure among public health experts, healthcare providers, clinicians, parents, families, patient advocates, and diverse social, ethnic and religious groups.

### Study setting

The study will be conducted at one of the sentinel hospitals of Karachi, the National Institute of Child Health (NICH), because of their well-established pediatric care protocols and willingness to participate in the study. NICH is a 320-bed tertiary care public sector hospital providing care to infants and children in Karachi. The FGDs will be conducted at the outpatient department (OPD) and well-baby clinics of the NICH hospital where families and relatives are visiting.

### Study participants

#### Key-informant interviews (KIIs)

We will invite ‘key informants’ such as the medical director-NICH, healthcare providers (doctors, nurses/ midwives), religious leaders, health sector representatives from the government, public health experts, obstetricians, neonatologists, members of the ethics review committee and professionals involved in proceedings related to death and dying (mortuary attendants/ body preparers) to understand their views and acceptability of the MITS procedure (Table 1). KIIs will be sent/emailed a letter inviting them to participate in the qualitative study. A few KIIs will be arranged at NICH and others will take place at locations preferred by the interviewees. Key informants will be requested to sign consent forms before the interview begins, in which they will agree that the interview can be audio-recorded and written notes can be taken by a note-taker to record interviewee expressions and statements. The key-informant interview will be later transcribed into the local language. However, no identifying characteristic will be included in the transcription. Initially, the KII will involve discussion around health status of pregnant women and their perceptions about neonatal death/stillbirths. Later, the discussion will move towards exploring views about causes of neonatal deaths/stillbirths and acceptability of MITS procedure among healthcare professionals. Finally, the interview will explore perceived facilitators and barriers for implementation of MITS procedure and health systems requirements for implementing the new method to determine CoD. We anticipate that 13–15 participants will be recruited for KIIs, but we will cease interviews once data saturation has been achieved.

**Table 1:** Study participants for KIIs and FGDs

	Sample range
Participants for Key-informant Interviews (KIIs)	
Medical director	01 KIIs
Healthcare providers at NICH (Doctors/ nurses/ midwives)	02–03 KIIs
Professionals involved in proceedings related to death and dying (e.g., mortuary attendants, body preparers)	01–02 KIIs
Religious leaders	01–03 KIIs
Health sector government representatives (Secretary Health, Sindh)	01 KIIs
Public Health Experts (representatives of key international NGOs)	01–03 KIIs
Clinicians (MCH specialists, obstetrician and neonatologist)	02 KIIs
Expert from Bioethics committee	01 KIIs
Participants for Focus Group Discussion (FGDs)	
Mothers of newborns who are visiting OPD and well-baby clinics of NICH hospital for regular post-natal check-ups	03
Fathers of newborns who are visiting OPD and well-baby clinics of NICH hospital for regular post-natal check-ups	03
Parents of newborns who are visiting OPD and well-baby clinics of NICH hospital for regular post-natal check-ups	02
Relatives of deceased (Aunts/ uncles)	02

#### Focus group discussions (FGDs)

FGDs will be conducted with the families and relatives of newborns who are visiting the OPD and well-baby clinics of NICH hospital for regular growth monitoring, post-natal check-ups and vaccinations. A few FGDs will be conducted with the relatives of families who have experienced a recent neonatal death/stillbirth. Considering the cultural and ethical sensitivity, the research will not have focus groups with parents who have experienced a recent neonatal death/stillbirth. Additionally, FGDs will not be conducted with the parents of admitted newborns who are waiting at the in-patient areas (Table 1). FGDs will be arranged in one of the meeting rooms at NICH. Focus groups will be facilitated by a trained moderator who is experienced in this area. Focus group participants will be requested to sign a consent form before the dialogue begins, in which case they will agree that the discussion can be noted and audio-recorded for transcription purpose. Participants will be assured that their anonymity will be maintained and no identifying features will be mentioned on the transcript. The major themes will include a general discussion about the health status of pregnant women, perceptions about neonatal death/stillbirths and related practices, views about causes of neonatal deaths and stillbirths, acceptability of MITS among parents and families, perceived facilitators and barriers for implementation of MITS, and exploring perceptions of parents/families who have experienced a prior loss or relatives of families who have been affected by the recent neonatal death/stillbirth. We anticipate that 8–10 FGDs will be conducted, with at least 6 participants in each one. However, FGDs will be ceased once data saturation has been reached.

### Eligibility criteria

The inclusion and exclusion criteria for study participants are provided below:

#### Inclusion criteria

- Parents and relatives of newborns who are visiting the OPD and well-baby clinics of NICH hospital for regular growth monitoring, post-natal check-ups and vaccinations.
- Relatives of families who have experienced a recent neonatal death/stillbirth.
- Key informants’ such as the medical director-NICH, healthcare providers (Doctors, nurses/ midwives), religious leaders, health sector representatives from the Government, public health expert, obstetrician, neonatologist, member of ethics review committee and professionals involved in proceedings related to death and dying (mortuary attendants’/ body preparers) who are willing to give consent to participate in the study.

#### Exclusion criteria

- We will not interview parents who have experienced a recent neonatal death/stillbirth.
- Considering the cultural and ethical sensitivity, this qualitative research will not interview parents of admitted newborns who are waiting in the in-patient areas.
- Participants (parents/families/KIIs) who are not willing to take part in this study.

### Ethical considerations

Study participants will be asked to provide informed, written consent prior to participation in this study. Participants who are unable to write their names will be asked to provide a thumbprint to symbolize their consent to participate. Ethical approval from NICH and Aga Khan University Ethical Review Committee (AKU-ERC) has been taken prior initiating this study.

### Data collection

Separate semi-structured interview guides have been developed for KIIs and FGDs. The interview guide will help explore participants’ views towards full autopsy and MITS, perceived benefits, potential limitations or concerns, and implementation into clinical practice. At the start of the interview, participants will be provided with a standardized overview of autopsy and MITS (Table 2).

**Table 2:** Overview of open autopsy and MITS

Full autopsy - Most comprehensive and complete method to estimate CoD - Rarely undertaken in such resource-poor environments due to cultural, financial, religious, and physical barriers - Very extensive examination of internal organs begins with the creation of a Y or U- shaped incision from both shoulders joining over the sternum and continuing down to the pubic bone	
MITS - The MITS procedure involves body inspection and recording of basic anthropometric data; body weight, height/length, mid-upper arm circumference, head circumference, lower leg length and foot length - The procedure involves body palpation by a MITS specialist. - The procedure involves imaging/photography by a MITS technician - The procedure uses biopsy needles to obtain samples of lung, brain, liver and other organs for histopathologic and microbiologic examination to help determine COD	

### Data analysis

The data will be transcribed into written-form from audio-recordings and will be analyzed via qualitative data analysis software NVivo 10. Written transcripts will be uploaded into NVivo 10 software to offer easy and organized retrieval of data for analysis. Thematic analysis will be carried out to analyze transcribed data collected through KIIs and FGDs. This involves an iterative process where data is coded, compared, contrasted and refined to generate emergent themes. Transcripts will be read several times to develop an interpretation of the participants’ perception regarding the acceptability of MITS. The transcribed text will be divided into ‘meaning units’ which will be later shortened and labeled with a ‘code’ without losing the study context. Codes will be then analyzed and grouped into categories to capture. In the final step, similar categories will be assembled under main themes. Two independent investigators will perform the coding, category creation, and thematic analyses, and discrepancies will be resolved to reduce researcher’s bias. To ensure the credibility of the research, study data will be triangulated by the data sources (parents, mothers, fathers, relatives, healthcare providers, clinicians, public health expert, bioethics expert) and data collection methods (FGDs and KIIs), to compare alternative perspective and reveal any inconsistencies [13].

## Discussion

This qualitative study will provide a unique opportunity, thorough insight into the views of families and healthcare professionals, towards the use of MITS, a less invasive autopsy procedure. Such in-depth insights will be crucial to develop an understanding of the cultural, religious and socio-behavioral factors that may facilitate or hinder the acceptability of the MITS procedure. The study will describe how parents view the MITS procedure, and how these views relate to socio-demographic factors such as culture, religion and socio-economic status. The study will also highlight how a less invasive autopsy can address some of the barriers, such as delay in carrying out funeral practices and concerns regarding organ and tissue removal, which are particularly significant for some cultural and religious groups. Finally, the findings will have significant implications for future practice and policy regarding the provision of post-mortem services.

## Abbreviations

CoD: Cause of death
ERC: Ethical Review Committee
FGDs: Focus group discussions
KIIs: Key-informant interviews
LMIC: Low-and-middle-income-countries
MCH: Maternal and child health
MITS: Minimal invasive tissue sampling
OPD: Outpatient department
SDG: Sustainable Development Goal
WHO: World Health Organization
